# Poly[[nona­aqua­bis­(μ-5-hy­droxy­benzene-1,3-di­carboxyl­ato)(5-hy­droxy­benzene-1,3-di­carboxyl­ato)dicerium(III)] hexa­hydrate]

**DOI:** 10.1107/S1600536814007727

**Published:** 2014-04-16

**Authors:** Xiao Fan, Carole Daiguebonne, Olivier Guillou, Magatte Camara

**Affiliations:** a INSA, UMR 6226, Institut des Sciences Chimiques de Rennes, 35 708 Rennes, France; b Universit Assane Seck de Ziguinchor, LCPM, BP 523 Ziguinchor, Senegal

## Abstract

In the title coordination polymer, {[Ce_2_(C_8_H_4_O_5_)_3_(H_2_O)_9_]·6H_2_O}_*n*_, the asymmetric unit is formed by two Ce^III^ atoms, three 5-hy­droxy­benzene-1,3-di­carboxyl­ate ligands, nine coordinating water mol­ecules and six water mol­ecules of crystallization. The two Ce^III^ atoms are bridged by 5-hy­droxy­benzene-1,3-di­carboxyl­ate ligands acting in a bis-bidentate coordination mode, generating infinite chains along [101]. Both independent metal atoms are nine-coordinated, one by four O atoms from the carboxyl­ate groups of two bridging 5-hy­droxy­benzene-1,3-di­carboxyl­ate ligands and five O atoms from water mol­ecules, generating a tricapped trigonal–prismatic geometry. The coordination around the second Ce^III^ atom is similar, except that one of the water mol­ecules is replaced by an O atom from an additional 5-hy­droxy­benzene-1,3-di­carboxyl­ate ligand acting in a monodentate coordination mode and forming a capped square-anti­prismatic geometry.

## Related literature   

For background to this field of research, see: Daiguebonne *et al.* (1998 ▶); Qiu *et al.* (2007 ▶); Eddaoudi *et al.* (2002 ▶); Kerbellec *et al.* (2008 ▶); Jeon & Clérac (2012 ▶); Calvez *et al.* (2008 ▶); Binnemans (2009 ▶); Daiguebonne *et al.* (2008 ▶); Freslon *et al.* (2014 ▶). For previously reported crystal structures that involve 5-hy­droxy­benzene-1,3-di­carboxyl­ate, see: Ermer & Neudörfl (2001 ▶); Lin *et al.* (2010 ▶); Xu & Li (2004 ▶); Chen *et al.* (2012 ▶); Huang *et al.* (2008 ▶). For details concerning the synthesis, see: Henisch & Rustum (1970 ▶); Henisch (1988 ▶); Daiguebonne *et al.* (2003 ▶).
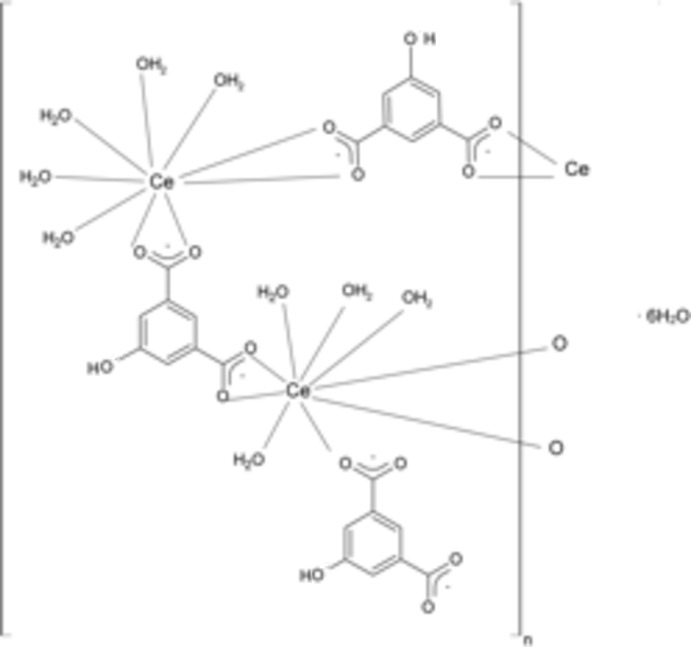


## Experimental   

### 

#### Crystal data  

[Ce_2_(C_8_H_4_O_5_)_3_(H_2_O)_9_]·6H_2_O
*M* *_r_* = 1090.82
Monoclinic, 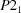

*a* = 10.7150 (3) Å
*b* = 11.1039 (2) Å
*c* = 16.3611 (4) Å
β = 100.975 (2)°
*V* = 1911.01 (8) Å^3^
*Z* = 2
Mo *K*α radiation
μ = 2.46 mm^−1^
*T* = 293 K
0.14 × 0.05 × 0.04 mm

#### Data collection  

Kappa CCD diffractometer
Absorption correction: multi-scan (Blessing, 1995 ▶) *T*_min_ = 0.763, *T*_max_ = 0.866
26639 measured reflections
8644 independent reflections
7711 reflections with *I* > 2σ(*I*)
*R*_int_ = 0.040

#### Refinement  

*R*[*F* ^2^ > 2σ(*F* ^2^)] = 0.035
*wR*(*F* ^2^) = 0.088
*S* = 1.06
8644 reflections
506 parameters
1 restraint
H-atom parameters constrained
Δρ_max_ = 1.46 e Å^−3^
Δρ_min_ = −1.28 e Å^−3^
Absolute structure: Flack (1983 ▶), 4150 Friedel pairs
Absolute structure parameter: 0.166 (19)

### 

Data collection: *COLLECT* (Nonius, 1998 ▶); cell refinement: *COLLECT*; data reduction: *EVALCCD* (Duisenberg *et al.*, 2003 ▶); program(s) used to solve structure: *SHELXS97* (Sheldrick, 2008 ▶); program(s) used to refine structure: *SHELXL97* (Sheldrick, 2008 ▶); molecular graphics: *DIAMOND* (Brandenburg, 2001 ▶); software used to prepare material for publication: *publCIF* (Westrip, 2010 ▶).

## Supplementary Material

Crystal structure: contains datablock(s) global, I. DOI: 10.1107/S1600536814007727/lr2124sup1.cif

Structure factors: contains datablock(s) I. DOI: 10.1107/S1600536814007727/lr2124Isup2.hkl

CCDC reference: 995942

Additional supporting information:  crystallographic information; 3D view; checkCIF report

## Figures and Tables

**Fig. 1.**
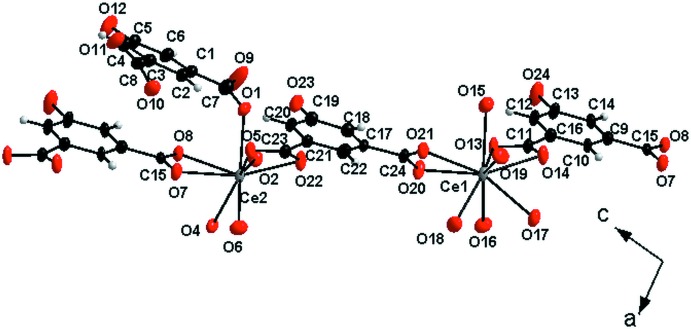
Extended asymmetric unit of the title compound. Displacement ellipsoids are drawn at a 50% probability level.

**Fig. 2.**
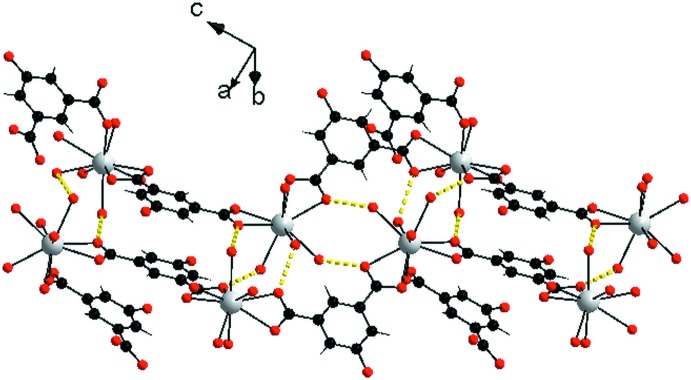
Projection view of molecular chains motif of {[Ce2(C8H4O5)3(H2O)9].6H2O}n. Yellow dotted lines symbolize assumed hydrogen-bonds (with inter-atomic distances between involved O atoms in the range 2.7–2.8 Å)

**Fig. 3.**
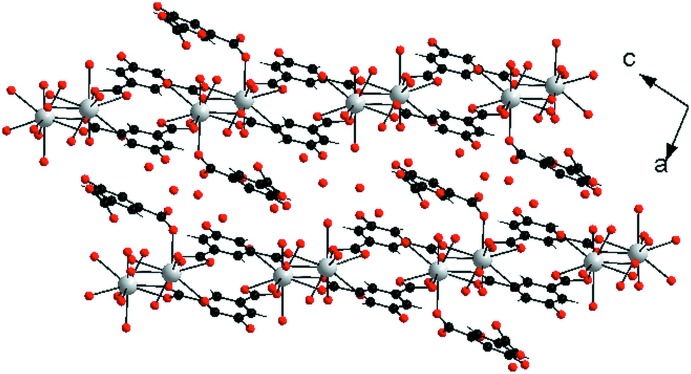
Projection view along the b axis of two neighboring double-chains molecular motifs of {[Ce2(C8H4O5)3(H2O)9].6H2O}n.

## supplementary crystallographic information

### 1. Introduction

For more than a decade, our group has been involved in the synthesis of benzene-poly-carboxyl­ate lanthanide-based coordination polymers: (Daiguebonne *et al.*, 1998), (Qiu *et al.*, 2007); because of their great inter­est in gas storage: (Eddaoudi *et al.* 2002), (Kerbellec *et al.*, 2008); molecular magnetism: (Jeon *et al.*, 2012), (Calvez *et al.*, 2008) or luminescence: (Binnemans, 2009), (Daiguebonne *et al.*, 2008). In the frame of this work we have recently proved that lanthanide-based coordination polymers can exhibit original luminescence properties when a donor group is present in the vicinity of the lanthanide ion: (Freslon *et al.*, 2014). Therefore we have undertaken the study of lanthanide-based coordination polymers that involves 5-hy­droxy­benzene-1,3-di­carboxyl­ate as ligand. This ligand has previously led to extended molecular networks in association with organic molecules: (Ermer & Neudörfl, 2001), transition metal ions: ( Lin *et al.*, 2010) or lanthanide ions: (Xu & Li , 2004), (Chen *et al.*, 2012), (Huang *et al.* , 2008). Previously reported lanthanide-based coordination polymers have been obtained by hydro­thermal methods. The structure described here has been obtained on the basis of single crystals that have grown in gel medium.

### 2.1. Synthesis and crystallization

5-Hy­droxy­benzene-1,3-di­carb­oxy­lic acid was purchased from Alfa Aesar and used without further purification. Its di-sodium salt was prepared by addition of two equivalent of sodium hydroxide to an aqueous suspension of the acid. Then the obtained clear solution was evaporated to dryness. The resulting solid was suspended in a small amount of ethanol. The mixture was stirred and refluxed for 1 hour. Upon addition of eth­oxy­ethane, precipitation occurred. After filtration and drying the white powder of the di-sodium salt was obtained in 90% yield.

Hydrated cerium chloride was purchased from A.M.P.E.R.E Industrie and used without further purification. Tetra­methyl­orthosilicate (TMOS) was purchased from Acros Organics and jellified according to established procedures: (Henisch, 1988),( Henisch & Rustum, 1970), (Daiguebonne *et al.* ,2003). Dilute aqueous solutions (0.1 mol.L^-1^) of cerium (III) chloride and di-sodium 5-hy­droxy­benzene-1,3-di-carboxyl­ate were allowed to slowly diffuse through gel media in U-shaped tubes. After few weeks needle-like single crystals were obtained in the tubes that have been filled with a 7.5% gel (expressed in weight percent).

### 2.2. Refinement

Crystal data, data collection and structure refinement details are summarized in Table 1.

H-atoms from water molecules have not been assigned and were thus not included in the refinement, but they were taken into account for the chemical formula sum, moiety, weight, as well as for the absorption coefficient and the number of electrons in the unit cell.

### 3. Results and discussion

The crystal structure of [Ce_2_(C_8_H_4_O_5_)_3_(H_2_O)_9_,6H_2_O]_∞_ can be described on the basis of chains molecular motifs that spread in the (a+c) direction. Each chain is constituted by an alternation of cerium ions bridged by 5-hy­droxy­benzene-1,3-di-carboxyl­ate ligands. There are two crystallographically independent cerium (III) ions in the asymmetric unit. Both are nine-coordinated. Ce1 is bound by four oxygen atoms from carboxyl­ate groups and five oxygen atoms from water molecules that form a tricapped trigonal prism. On the other hand, Ce2 is bound by five oxygen atoms from carboxyl­ate groups and four oxygen atoms from water molecules that form a capped square anti­prism. There are three crystallographically independent ligands in the asymmetric unit. Two out of the three bridge the metal ions in a bis-bidentate manner. A third ligand is only linked to the Ce2 atom in a monodentate fashion. Its second carboxyl­ate clip is not bound and point toward the inter-molecular motifs space (Figure 1). This is in agreement with the IR spectrum that shows no characteristic peak of any protonated carboxyl­ate group.

The short distances ( in the range 2.7–2.8 Å) between some oxygen atoms allow to assume that neighboring chains are held together by strong inter­molecular hydrogen bond inter­actions forming a double-chains molecular motif (Figure 2). Ligands that are bound in a unidentate fashion are pointing between the double-chains molecular motifs. Oxygen atoms from the free carboxyl­ate clip are involved, with coordination and crystallization water molecules, in a complex Hydrogen-bonds network that ensure the stability of the crystal packing.

### Crystal data

**Table:**

[Ce_2_(C_8_H_4_O_5_)_3_(H_2_O)_9_]·6H_2_O	*F*(000) = 1084
*M**_r_* = 1090.82	*D*_x_ = 1.896 Mg m^−^^3^
Monoclinic, *P*2_1_	Mo *K*α radiation, λ = 0.71073 Å
Hall symbol: P 2yb	Cell parameters from 22389 reflections
*a* = 10.7150 (3) Å	θ = 2.9–27.5°
*b* = 11.1039 (2) Å	µ = 2.46 mm^−^^1^
*c* = 16.3611 (4) Å	*T* = 293 K
β = 100.975 (2)°	Needle, colourless
*V* = 1911.01 (8) Å^3^	0.14 × 0.05 × 0.04 mm
*Z* = 2	

### Data collection

**Table:**

Kappa CCD diffractometer	8644 independent reflections
Radiation source: Mo	7711 reflections with *I* > 2σ(*I*)
Graphite monochromator	*R*_int_ = 0.040
φ– and ω– scans	θ_max_ = 27.5°, θ_min_ = 3.1°
Absorption correction: multi-scan (Blessing,1995)	*h* = −13→13
*T*_min_ = 0.763, *T*_max_ = 0.866	*k* = −14→14
26639 measured reflections	*l* = −21→21

### Refinement

**Table:**

Refinement on *F*^2^	Secondary atom site location: difference Fourier map
Least-squares matrix: full	Hydrogen site location: inferred from neighbouring sites
*R*[*F*^2^ > 2σ(*F*^2^)] = 0.035	H-atom parameters constrained
*wR*(*F*^2^) = 0.088	*w* = 1/[σ^2^(*F*_o_^2^) + (0.0416*P*)^2^ + 2.8811*P*] where *P* = (*F*_o_^2^ + 2*F*_c_^2^)/3
*S* = 1.06	(Δ/σ)_max_ = 0.001
8644 reflections	Δρ_max_ = 1.46 e Å^−^^3^
506 parameters	Δρ_min_ = −1.28 e Å^−^^3^
1 restraint	Absolute structure: Flack (1983), 4150 Friedel pairs
Primary atom site location: structure-invariant direct methods	Absolute structure parameter: 0.166 (19)

### Special details

**Table:**

Geometry. All esds (except the esd in the dihedral angle between two l.s. planes) are estimated using the full covariance matrix. The cell esds are taken into account individually in the estimation of esds in distances, angles and torsion angles; correlations between esds in cell parameters are only used when they are defined by crystal symmetry. An approximate (isotropic) treatment of cell esds is used for estimating esds involving l.s. planes.
Refinement. Refinement of *F*^2^ against ALL reflections. The weighted *R*-factor *wR* and goodness of fit *S* are based on *F*^2^, conventional *R*-factors *R* are based on *F*, with *F* set to zero for negative *F*^2^. The threshold expression of *F*^2^ > σ(*F*^2^) is used only for calculating *R*-factors(gt) *etc*. and is not relevant to the choice of reflections for refinement. *R*-factors based on *F*^2^ are statistically about twice as large as those based on *F*, and *R*- factors based on ALL data will be even larger.

### Fractional atomic coordinates and isotropic or equivalent isotropic displacement parameters (Å^2^)

**Table:**

	*x*	*y*	*z*	*U*_iso_*/*U*_eq_	
Ce01	0.41401 (3)	0.24388 (2)	0.933831 (18)	0.02183 (10)	
Ce02	−0.09386 (3)	−0.17680 (2)	0.436383 (18)	0.01951 (9)	
O1	−0.2917 (4)	−0.1863 (6)	0.4899 (3)	0.0289 (10)	
O2	−0.1739 (6)	−0.3897 (5)	0.4199 (4)	0.0294 (13)	
O3	−0.0651 (4)	−0.2416 (6)	0.2877 (3)	0.0297 (11)	
O4	0.1035 (5)	−0.3002 (4)	0.4468 (3)	0.0284 (11)	
O5	−0.1871 (5)	0.0358 (5)	0.4380 (3)	0.0273 (12)	
O6	0.0564 (5)	−0.0265 (5)	0.3904 (4)	0.0352 (13)	
O7	0.0223 (5)	−0.0881 (5)	0.5719 (3)	0.0288 (12)	
O8	−0.0331 (4)	−0.2759 (5)	0.5849 (3)	0.0240 (11)	
O9	−0.3621 (7)	−0.3564 (5)	0.5332 (3)	0.076 (2)	
O10	−0.1659 (5)	0.1474 (5)	0.7003 (3)	0.0383 (11)	
O11	−0.2278 (5)	0.1263 (5)	0.8207 (3)	0.0458 (12)	
O12	−0.2563 (6)	−0.3228 (6)	0.8491 (3)	0.0482 (15)	
HO12	−0.2724	−0.3917	0.8324	0.072*	
O13	0.3158 (5)	0.0346 (5)	0.9375 (3)	0.0313 (13)	
O14	0.2395 (5)	0.1407 (5)	0.8273 (3)	0.0317 (12)	
O15	0.2116 (5)	0.2613 (7)	0.9923 (3)	0.0389 (14)	
O16	0.5582 (5)	0.0896 (5)	0.8858 (3)	0.0355 (13)	
O17	0.4364 (5)	0.3130 (7)	0.7868 (3)	0.0346 (12)	
O18	0.6140 (5)	0.3617 (5)	0.9463 (3)	0.0369 (13)	
O19	0.3268 (6)	0.4551 (6)	0.9146 (4)	0.0363 (15)	
O20	0.5302 (5)	0.1566 (5)	1.0706 (3)	0.0321 (12)	
O21	0.4675 (5)	0.3431 (5)	1.0802 (3)	0.0275 (12)	
O22	0.7420 (5)	−0.0683 (5)	1.3243 (3)	0.0334 (13)	
O23	0.5883 (5)	0.4408 (5)	1.3876 (3)	0.0323 (11)	
HO23	0.5524	0.4941	1.3573	0.048*	
O24	0.0790 (6)	−0.3571 (6)	0.8987 (3)	0.0546 (18)	
HO24	0.0427	−0.4137	0.8720	0.082*	
C1	−0.2907 (5)	−0.2074 (5)	0.6356 (3)	0.0264 (11)	
C2	−0.2621 (6)	−0.0885 (7)	0.6513 (4)	0.0267 (13)	
H2	−0.2582	−0.0364	0.6073	0.032*	
C3	−0.2387 (5)	−0.0449 (5)	0.7330 (4)	0.0287 (12)	
C4	−0.2354 (7)	−0.1267 (7)	0.7985 (5)	0.0329 (16)	
H4	−0.2159	−0.0998	0.8533	0.039*	
C5	−0.2607 (6)	−0.2469 (6)	0.7822 (4)	0.0321 (12)	
C6	−0.2887 (7)	−0.2889 (7)	0.7012 (4)	0.0299 (16)	
H6	−0.3060	−0.3700	0.6905	0.036*	
C7	−0.3179 (6)	−0.2537 (6)	0.5475 (4)	0.0343 (13)	
C8	−0.2092 (5)	0.0862 (5)	0.7522 (4)	0.0296 (12)	
C9	0.0619 (5)	−0.1745 (9)	0.7086 (4)	0.0236 (12)	
C10	0.1232 (6)	−0.0678 (7)	0.7441 (4)	0.0221 (15)	
H10	0.1332	−0.0023	0.7105	0.026*	
C11	0.1682 (7)	−0.0623 (7)	0.8300 (4)	0.0252 (15)	
C12	0.1521 (6)	−0.1620 (8)	0.8803 (4)	0.0292 (16)	
H12	0.1823	−0.1588	0.9375	0.035*	
C13	0.0919 (7)	−0.2634 (9)	0.8451 (4)	0.0306 (15)	
C14	0.0474 (6)	−0.2704 (8)	0.7594 (4)	0.0270 (15)	
H14	0.0076	−0.3403	0.7364	0.032*	
C15	0.0151 (5)	−0.1807 (8)	0.6173 (3)	0.0198 (11)	
C16	0.2435 (6)	0.0420 (7)	0.8667 (4)	0.0236 (14)	
C17	0.5721 (5)	0.2448 (8)	1.2077 (4)	0.0210 (12)	
C18	0.5608 (6)	0.3459 (6)	1.2559 (4)	0.0247 (15)	
H18	0.5258	0.4165	1.2310	0.030*	
C19	0.6018 (6)	0.3407 (7)	1.3407 (4)	0.0248 (14)	
C20	0.6592 (6)	0.2376 (8)	1.3782 (4)	0.0251 (13)	
H20	0.6879	0.2348	1.4355	0.030*	
C21	0.6734 (6)	0.1383 (6)	1.3289 (4)	0.0218 (14)	
C22	0.6282 (7)	0.1436 (7)	1.2435 (5)	0.0279 (17)	
H22	0.6365	0.0769	1.2106	0.033*	
C23	0.7462 (6)	0.0291 (6)	1.3655 (4)	0.0228 (14)	
C24	0.5212 (5)	0.2489 (8)	1.1145 (4)	0.0226 (12)	
O031	0.5264 (5)	0.4338 (5)	0.5427 (3)	0.0466 (12)	
O039	0.4961 (6)	0.1825 (6)	0.6563 (4)	0.0587 (16)	
O040	0.9555 (7)	0.2265 (8)	0.9363 (4)	0.085 (2)	
O041	0.7364 (5)	0.3758 (5)	0.6657 (3)	0.0506 (13)	
O043	0.0116 (9)	0.4262 (7)	0.8386 (5)	0.087 (3)	
O062	0.6940 (12)	0.4496 (7)	0.8113 (5)	0.129 (5)	

### Atomic displacement parameters (Å^2^)

**Table:**

	*U*^11^	*U*^22^	*U*^33^	*U*^12^	*U*^13^	*U*^23^
Ce01	0.0301 (2)	0.0174 (2)	0.01549 (19)	−0.00197 (15)	−0.00223 (15)	0.00039 (15)
Ce02	0.02548 (18)	0.01640 (19)	0.01496 (18)	0.00154 (14)	−0.00042 (14)	−0.00006 (14)
O1	0.034 (2)	0.033 (3)	0.021 (2)	0.003 (2)	0.0084 (19)	0.005 (3)
O2	0.038 (3)	0.018 (3)	0.031 (3)	0.001 (2)	0.004 (2)	−0.001 (2)
O3	0.034 (2)	0.034 (3)	0.018 (2)	0.001 (3)	−0.0010 (19)	−0.004 (3)
O4	0.033 (2)	0.032 (3)	0.020 (2)	0.0107 (18)	0.004 (2)	0.0049 (19)
O5	0.032 (3)	0.024 (3)	0.021 (3)	0.003 (2)	−0.006 (2)	0.001 (2)
O6	0.042 (3)	0.026 (3)	0.042 (3)	−0.001 (2)	0.016 (3)	−0.001 (2)
O7	0.041 (3)	0.024 (3)	0.018 (2)	−0.005 (2)	−0.001 (2)	0.004 (2)
O8	0.033 (2)	0.018 (3)	0.019 (2)	−0.003 (2)	−0.0007 (19)	−0.002 (2)
O9	0.139 (6)	0.057 (4)	0.039 (3)	−0.063 (4)	0.034 (3)	−0.019 (3)
O10	0.044 (3)	0.038 (3)	0.034 (2)	−0.013 (2)	0.012 (2)	−0.002 (2)
O11	0.059 (3)	0.050 (3)	0.033 (2)	−0.021 (2)	0.021 (2)	−0.015 (2)
O12	0.078 (4)	0.038 (3)	0.030 (3)	−0.001 (3)	0.013 (3)	0.012 (2)
O13	0.043 (3)	0.025 (3)	0.020 (3)	−0.006 (2)	−0.011 (2)	0.007 (2)
O14	0.042 (3)	0.026 (3)	0.022 (3)	−0.005 (2)	−0.008 (2)	0.006 (2)
O15	0.043 (3)	0.045 (4)	0.029 (3)	−0.003 (3)	0.008 (2)	−0.002 (3)
O16	0.048 (3)	0.026 (3)	0.036 (3)	0.002 (2)	0.016 (3)	−0.002 (2)
O17	0.046 (3)	0.036 (3)	0.020 (2)	−0.003 (3)	0.000 (2)	0.001 (3)
O18	0.039 (3)	0.039 (3)	0.033 (3)	−0.012 (2)	0.006 (2)	−0.010 (2)
O19	0.036 (3)	0.028 (3)	0.044 (4)	0.009 (2)	0.006 (3)	0.010 (3)
O20	0.052 (3)	0.019 (3)	0.021 (3)	0.010 (2)	−0.003 (2)	−0.003 (2)
O21	0.036 (3)	0.023 (3)	0.021 (2)	0.005 (2)	−0.002 (2)	0.002 (2)
O22	0.046 (3)	0.026 (3)	0.023 (3)	0.009 (2)	−0.008 (2)	−0.007 (2)
O23	0.047 (3)	0.025 (2)	0.023 (2)	0.010 (2)	0.003 (2)	−0.0036 (19)
O24	0.085 (4)	0.044 (4)	0.029 (3)	−0.033 (3)	−0.005 (3)	0.015 (3)
C1	0.028 (3)	0.028 (3)	0.024 (3)	−0.001 (2)	0.009 (2)	0.000 (2)
C2	0.028 (3)	0.033 (4)	0.018 (3)	−0.007 (3)	0.003 (2)	−0.007 (2)
C3	0.030 (3)	0.029 (3)	0.028 (3)	−0.002 (2)	0.008 (2)	0.000 (2)
C4	0.037 (4)	0.035 (4)	0.027 (3)	−0.005 (3)	0.008 (3)	−0.002 (3)
C5	0.038 (3)	0.035 (3)	0.024 (3)	0.000 (3)	0.009 (2)	0.006 (2)
C6	0.038 (4)	0.028 (4)	0.025 (3)	−0.004 (3)	0.010 (3)	0.000 (3)
C7	0.043 (3)	0.030 (3)	0.031 (3)	−0.002 (3)	0.010 (3)	−0.005 (3)
C8	0.030 (3)	0.030 (3)	0.031 (3)	−0.006 (2)	0.011 (2)	−0.007 (2)
C9	0.025 (3)	0.025 (3)	0.019 (3)	−0.003 (3)	0.001 (2)	−0.004 (4)
C10	0.026 (3)	0.023 (4)	0.014 (3)	−0.007 (3)	−0.005 (3)	−0.002 (3)
C11	0.028 (3)	0.025 (4)	0.022 (3)	−0.005 (3)	0.001 (3)	0.001 (3)
C12	0.036 (3)	0.034 (4)	0.015 (3)	−0.012 (3)	−0.002 (3)	−0.008 (3)
C13	0.040 (4)	0.034 (4)	0.016 (3)	−0.012 (4)	0.000 (3)	0.006 (3)
C14	0.026 (3)	0.033 (4)	0.019 (3)	−0.005 (3)	−0.005 (2)	−0.002 (3)
C15	0.017 (2)	0.025 (3)	0.016 (3)	0.000 (3)	−0.001 (2)	0.000 (3)
C16	0.028 (3)	0.024 (4)	0.017 (3)	−0.003 (3)	−0.001 (3)	0.000 (3)
C17	0.023 (3)	0.025 (3)	0.014 (3)	0.000 (3)	0.001 (2)	0.001 (3)
C18	0.030 (3)	0.016 (4)	0.028 (3)	0.003 (2)	0.005 (3)	0.003 (3)
C19	0.032 (3)	0.016 (3)	0.026 (3)	−0.001 (3)	0.004 (3)	0.000 (3)
C20	0.031 (3)	0.028 (3)	0.015 (3)	−0.001 (3)	0.003 (2)	−0.011 (3)
C21	0.024 (3)	0.021 (4)	0.020 (3)	−0.001 (2)	0.003 (3)	0.005 (3)
C22	0.037 (4)	0.021 (4)	0.026 (4)	0.002 (3)	0.008 (3)	−0.005 (3)
C23	0.028 (3)	0.019 (4)	0.020 (3)	0.002 (3)	0.002 (3)	0.003 (3)
C24	0.030 (3)	0.021 (3)	0.015 (3)	0.000 (3)	0.001 (2)	0.002 (3)
O031	0.053 (3)	0.040 (3)	0.048 (3)	−0.014 (2)	0.011 (2)	−0.002 (2)
O039	0.073 (4)	0.048 (4)	0.050 (3)	−0.007 (3)	−0.001 (3)	−0.003 (3)
O040	0.071 (4)	0.132 (7)	0.054 (4)	−0.040 (5)	0.018 (3)	−0.030 (4)
O041	0.056 (3)	0.044 (3)	0.054 (3)	−0.004 (2)	0.018 (3)	−0.006 (3)
O043	0.145 (7)	0.058 (5)	0.062 (4)	−0.016 (4)	0.028 (5)	−0.019 (4)
O062	0.292 (14)	0.054 (5)	0.071 (5)	−0.026 (6)	0.112 (7)	−0.005 (4)

### Geometric parameters (Å, º)

**Table:**

Ce01—O18	2.486 (5)	O23—HO23	0.8200
Ce01—O19	2.522 (6)	O24—C13	1.385 (9)
Ce01—O16	2.530 (5)	O24—HO24	0.8200
Ce01—O20	2.537 (5)	C1—C2	1.368 (9)
Ce01—O15	2.539 (5)	C1—C6	1.401 (9)
Ce01—O13	2.556 (6)	C1—C7	1.506 (8)
Ce01—O14	2.570 (5)	C2—C3	1.400 (8)
Ce01—O17	2.579 (5)	C2—H2	0.9300
Ce01—O21	2.598 (5)	C3—C4	1.400 (9)
Ce01—C24	2.961 (6)	C3—C8	1.510 (8)
Ce01—C16	2.967 (7)	C4—C5	1.378 (10)
Ce02—O1	2.445 (4)	C4—H4	0.9300
Ce02—O4	2.498 (5)	C5—C6	1.382 (10)
Ce02—O2	2.512 (6)	C6—H6	0.9300
Ce02—O7	2.527 (5)	C9—C14	1.378 (11)
Ce02—O6	2.531 (5)	C9—C10	1.425 (11)
Ce02—O5	2.566 (6)	C9—C15	1.484 (8)
Ce02—O22^i^	2.584 (5)	C10—C11	1.397 (9)
Ce02—O3	2.610 (5)	C10—H10	0.9300
Ce02—O8	2.633 (5)	C11—C12	1.410 (11)
Ce02—C23^i^	2.958 (7)	C11—C16	1.473 (10)
Ce02—C15	2.968 (6)	C12—C13	1.369 (11)
O1—C7	1.276 (8)	C12—H12	0.9300
O5—C23^i^	1.265 (8)	C13—C14	1.394 (8)
O7—C15	1.279 (9)	C14—H14	0.9300
O8—C15	1.250 (10)	C17—C22	1.353 (11)
O9—C7	1.240 (8)	C17—C18	1.391 (10)
O10—C8	1.243 (7)	C17—C24	1.519 (8)
O11—C8	1.257 (7)	C18—C19	1.374 (9)
O12—C5	1.375 (8)	C18—H18	0.9300
O12—HO12	0.8200	C19—C20	1.386 (11)
O13—C16	1.268 (8)	C20—C21	1.391 (10)
O14—C16	1.267 (9)	C20—H20	0.9300
O20—C24	1.265 (10)	C21—C22	1.391 (10)
O21—C24	1.272 (9)	C21—C23	1.503 (9)
O22—C23	1.271 (8)	C22—H22	0.9300
O22—Ce02^ii^	2.584 (5)	C23—O5^ii^	1.265 (8)
O23—C19	1.375 (9)	C23—Ce02^ii^	2.958 (7)
			
O18—Ce01—O19	79.28 (19)	O7—Ce02—C15	25.3 (2)
O18—Ce01—O16	79.28 (18)	O6—Ce02—C15	98.89 (19)
O19—Ce01—O16	145.56 (18)	O5—Ce02—C15	94.8 (2)
O18—Ce01—O20	81.84 (18)	O22^i^—Ce02—C15	143.09 (18)
O19—Ce01—O20	125.0 (2)	O3—Ce02—C15	146.23 (16)
O16—Ce01—O20	77.81 (17)	O8—Ce02—C15	24.9 (2)
O18—Ce01—O15	135.56 (19)	C23^i^—Ce02—C15	119.4 (2)
O19—Ce01—O15	69.7 (2)	C7—O1—Ce02	128.1 (4)
O16—Ce01—O15	141.7 (2)	C23^i^—O5—Ce02	95.0 (4)
O20—Ce01—O15	90.47 (18)	C15—O7—Ce02	97.0 (4)
O18—Ce01—O13	145.98 (19)	C15—O8—Ce02	92.7 (4)
O19—Ce01—O13	134.74 (16)	C5—O12—HO12	109.5
O16—Ce01—O13	70.74 (18)	C16—O13—Ce01	95.9 (4)
O20—Ce01—O13	76.54 (18)	C16—O14—Ce01	95.2 (4)
O15—Ce01—O13	71.1 (2)	C24—O20—Ce01	96.5 (4)
O18—Ce01—O14	142.62 (17)	C24—O21—Ce01	93.4 (4)
O19—Ce01—O14	97.2 (2)	C23—O22—Ce02^ii^	94.0 (4)
O16—Ce01—O14	84.03 (18)	C19—O23—HO23	109.5
O20—Ce01—O14	126.83 (18)	C13—O24—HO24	109.5
O15—Ce01—O14	74.10 (18)	C2—C1—C6	120.6 (6)
O13—Ce01—O14	50.29 (16)	C2—C1—C7	120.3 (5)
O18—Ce01—O17	71.82 (18)	C6—C1—C7	119.0 (5)
O19—Ce01—O17	72.9 (2)	C1—C2—C3	120.4 (6)
O16—Ce01—O17	75.00 (19)	C1—C2—H2	119.8
O20—Ce01—O17	144.98 (17)	C3—C2—H2	119.8
O15—Ce01—O17	124.49 (18)	C2—C3—C4	118.7 (6)
O13—Ce01—O17	113.84 (19)	C2—C3—C8	121.6 (6)
O14—Ce01—O17	71.64 (16)	C4—C3—C8	119.6 (6)
O18—Ce01—O21	70.48 (16)	C5—C4—C3	120.4 (7)
O19—Ce01—O21	74.45 (19)	C5—C4—H4	119.8
O16—Ce01—O21	122.17 (18)	C3—C4—H4	119.8
O20—Ce01—O21	50.52 (16)	O12—C5—C4	117.7 (6)
O15—Ce01—O21	71.05 (16)	O12—C5—C6	121.6 (6)
O13—Ce01—O21	112.54 (17)	C4—C5—C6	120.7 (7)
O14—Ce01—O21	144.92 (16)	C5—C6—C1	119.0 (6)
O17—Ce01—O21	133.6 (2)	C5—C6—H6	120.5
O18—Ce01—C24	75.15 (18)	C1—C6—H6	120.5
O19—Ce01—C24	99.8 (2)	O9—C7—O1	122.0 (6)
O16—Ce01—C24	100.3 (2)	O9—C7—C1	119.5 (6)
O20—Ce01—C24	25.1 (2)	O1—C7—C1	118.5 (6)
O15—Ce01—C24	79.62 (16)	O10—C8—O11	123.9 (6)
O13—Ce01—C24	94.4 (2)	O10—C8—C3	118.4 (5)
O14—Ce01—C24	141.20 (18)	O11—C8—C3	117.6 (5)
O17—Ce01—C24	146.94 (18)	C14—C9—C10	119.5 (6)
O21—Ce01—C24	25.4 (2)	C14—C9—C15	121.1 (7)
O18—Ce01—C16	152.66 (17)	C10—C9—C15	119.4 (7)
O19—Ce01—C16	118.0 (2)	C11—C10—C9	119.4 (7)
O16—Ce01—C16	75.10 (19)	C11—C10—H10	120.3
O20—Ce01—C16	101.68 (19)	C9—C10—H10	120.3
O15—Ce01—C16	71.77 (19)	C10—C11—C12	119.7 (7)
O13—Ce01—C16	25.16 (18)	C10—C11—C16	120.1 (6)
O14—Ce01—C16	25.18 (17)	C12—C11—C16	119.9 (6)
O17—Ce01—C16	92.26 (19)	C13—C12—C11	120.0 (6)
O21—Ce01—C16	132.36 (17)	C13—C12—H12	120.0
C24—Ce01—C16	118.6 (2)	C11—C12—H12	120.0
O1—Ce02—O4	137.07 (18)	C12—C13—O24	116.6 (6)
O1—Ce02—O2	72.3 (2)	C12—C13—C14	120.9 (7)
O4—Ce02—O2	76.04 (18)	O24—C13—C14	122.6 (7)
O1—Ce02—O7	91.05 (17)	C9—C14—C13	120.5 (7)
O4—Ce02—O7	83.36 (17)	C9—C14—H14	119.8
O2—Ce02—O7	124.20 (19)	C13—C14—H14	119.8
O1—Ce02—O6	141.2 (2)	O8—C15—O7	120.0 (5)
O4—Ce02—O6	78.68 (17)	O8—C15—C9	119.9 (7)
O2—Ce02—O6	144.32 (17)	O7—C15—C9	120.1 (7)
O7—Ce02—O6	76.65 (17)	O8—C15—Ce02	62.4 (3)
O1—Ce02—O5	70.7 (2)	O7—C15—Ce02	57.7 (3)
O4—Ce02—O5	146.11 (17)	C9—C15—Ce02	175.2 (6)
O2—Ce02—O5	137.85 (14)	O14—C16—O13	118.4 (6)
O7—Ce02—O5	76.11 (18)	O14—C16—C11	120.6 (6)
O6—Ce02—O5	70.57 (17)	O13—C16—C11	120.9 (6)
O1—Ce02—O22^i^	75.81 (17)	O14—C16—Ce01	59.6 (4)
O4—Ce02—O22^i^	139.21 (15)	O13—C16—Ce01	59.0 (4)
O2—Ce02—O22^i^	100.97 (19)	C11—C16—Ce01	174.9 (5)
O7—Ce02—O22^i^	126.57 (17)	C22—C17—C18	120.3 (6)
O6—Ce02—O22^i^	82.36 (19)	C22—C17—C24	120.3 (7)
O5—Ce02—O22^i^	50.55 (16)	C18—C17—C24	119.5 (7)
O1—Ce02—O3	125.89 (16)	C19—C18—C17	119.5 (6)
O4—Ce02—O3	70.36 (16)	C19—C18—H18	120.2
O2—Ce02—O3	74.88 (19)	C17—C18—H18	120.2
O7—Ce02—O3	143.05 (16)	C18—C19—O23	118.8 (6)
O6—Ce02—O3	73.14 (18)	C18—C19—C20	120.7 (6)
O5—Ce02—O3	112.25 (18)	O23—C19—C20	120.4 (6)
O22^i^—Ce02—O3	69.68 (16)	C19—C20—C21	119.1 (6)
O1—Ce02—O8	74.37 (15)	C19—C20—H20	120.4
O4—Ce02—O8	69.52 (14)	C21—C20—H20	120.4
O2—Ce02—O8	74.04 (18)	C20—C21—C22	119.6 (7)
O7—Ce02—O8	50.16 (16)	C20—C21—C23	120.9 (6)
O6—Ce02—O8	119.37 (18)	C22—C21—C23	119.3 (6)
O5—Ce02—O8	113.79 (16)	C17—C22—C21	120.7 (7)
O22^i^—Ce02—O8	149.85 (16)	C17—C22—H22	119.6
O3—Ce02—O8	133.78 (18)	C21—C22—H22	119.6
O1—Ce02—C23^i^	72.35 (19)	O5^ii^—C23—O22	120.3 (6)
O4—Ce02—C23^i^	150.49 (16)	O5^ii^—C23—C21	118.9 (6)
O2—Ce02—C23^i^	121.66 (19)	O22—C23—C21	120.8 (6)
O7—Ce02—C23^i^	101.21 (19)	O5^ii^—C23—Ce02^ii^	59.8 (4)
O6—Ce02—C23^i^	74.19 (18)	O22—C23—Ce02^ii^	60.6 (4)
O5—Ce02—C23^i^	25.21 (18)	C21—C23—Ce02^ii^	176.0 (4)
O22^i^—Ce02—C23^i^	25.37 (17)	O20—C24—O21	119.5 (5)
O3—Ce02—C23^i^	90.60 (18)	O20—C24—C17	119.6 (7)
O8—Ce02—C23^i^	135.04 (16)	O21—C24—C17	120.9 (7)
O1—Ce02—C15	81.10 (14)	O20—C24—Ce01	58.3 (3)
O4—Ce02—C15	75.91 (17)	O21—C24—Ce01	61.2 (3)
O2—Ce02—C15	98.9 (2)	C17—C24—Ce01	176.7 (6)

Symmetry codes: (i) *x*−1, *y*, *z*−1; (ii) *x*+1, *y*, *z*+1.
